# The senescence-associated secretory phenotype (SASP) from mesenchymal stromal cells impairs growth of immortalized prostate cells but has no effect on metastatic prostatic cancer cells

**DOI:** 10.18632/aging.102172

**Published:** 2019-08-14

**Authors:** Nicola Alessio, Domenico Aprile, Tiziana Squillaro, Giovanni Di Bernardo, Mauro Finicelli, Mariarosa AB Melone, Gianfranco Peluso, Umberto Galderisi

**Affiliations:** 1 Department of Experimental Medicine, “Luigi Vanvitelli” Campania University, Naples, Italy; 2 Sbarro Institute for Cancer Research and Molecular Medicine, Center for Biotechnology, Temple University, Philadelphia, PA 19107, USA; 3 Institute for Research on Terrestrial Ecosystems (IRET), CNR, Naples, Italy; 4 Department of Advanced Medical and Surgical Sciences, “Luigi Vanvitelli” Campania University, Naples, Italy; 5 Inter-University Neuroscience Research Center (CIRN), Naples, Italy

**Keywords:** mesenchymal stem cells, senescence, secretome

## Abstract

Senescent cells secrete inflammatory cytokines, proteases, and other factors, which are indicated as senescence-associated secretory phenotype (SASP). There are contrasting studies on the role of the SASP in cancer. Studies suggested that cancer cells may misuse the senescent secretome for their growth. Other investigations evidenced that the SASP may induce cancer growth arrest, senescence, or apoptosis. These conflicting data can be reconciled considering that cancer cells can coax senescent cells to secrete factors for their survival, thus abrogating the SASP’s anti-cancer effect. Cancer stage may also have an impact on the capacity of the SASP to block tumor proliferation and promote senescence. Indeed, senescence is associated with a permanent cell cycle arrest, which needs functional cell cycle checkpoints. We evaluated the SASP effect on the *in vitro* biological properties of PNT2 and PC3 cells, which are immortalized prostate cells and metastatic prostatic cancer cells, respectively. We evidenced that SASPs, coming either from mesenchymal stromal cells treated with H_2_0_2_ or with low X-ray doses, induced senescence of immortalized cells but not of cancer cells. Hence, the SASP released by acute senescent cells should be considered as an effective weapon against pre-tumorigenesis events rather than an anti-cancer mechanism acting on malignant cells.

## Introduction

Genomic stress, induced by DNA damage, telomere shortening, extensive mitogenic signals, and changes in chromatin organization, can trigger cellular senescence, which stops cell division and causes loss of the cell’s functions. Senescent cells present some specific features, such as enlarged and flattened morphology, high β-galactosidase activity, senescence-associated heterochromatin foci, and changes in gene expression. Senescent cells secrete several inflammatory cytokines, growth factors, proteases, and other factors, which collectively are indicated as the senescence-associated secretory phenotype (SASP). Senescence is not static but is a progressive process that starts from a pre-senescent status to reach full senescence. The turning point is the passage from early to full senescence when cells proceed from transient to a stable cell-cycle arrest that is sustained by P16-RB and/or P53–P21 pathways. The progression to full senescence is accompanied by chromatin remodeling and SASP production, whose composition may change with time [1,2].

The above-described events indicate that senescence is a complex phenomenon and is difficult to study. Moreover, there are different types of senescent cells that may have peculiar properties besides a common background of shared features. There are two classes of senescent cells: acute and chronic. Extrinsic stressors (chemical and physical agents) may induce acute senescence whereas persistent cellular stress, such as extended proliferation with associated DNA replication and accumulation of genomic damages, may trigger chronic senescence, also named replicative senescence. Different stressors trigger specific acute senescent phenotypes. Indeed, in a previous finding, we demonstrated that oxidative stress, doxorubicin treatment, and X-ray irradiation induced acute senescent phenotypes that differ in their metabolic needs, autophagy status and SASP components. These phenotypes, however, share the typical feature of senescent condition [3,4]. These commonalities and differences between senescent phenotypes explain how cellular senescence could be involved in several biological processes, such as tumor suppression, tumor promotion, tissue repair, development, and aging [1,2].

Senescent cells can use the SASP to accomplish several tasks. The SASP released by damaged cells can promote surrounding cells to senesce, thus aiding minimally DNA-damaged cells to enter senescence. Some factors present in the SASP attract and activate immune system cells to dispose senescent cells. The SASP may also alter tissue and organ functions, thus contributing to individual aging [1,2].

There are contrasting studies on the role of the SASP in cancer. Many studies suggest that cancer cells may misuse the senescent secretome. Some investigations showed that the SASP released by senescent fibroblasts may promote cancer [5–10]. Nevertheless, there are other studies evidencing that the secretome of senescent fibroblasts may induce growth arrest and apoptosis of cancer cells [11]. These conflicting investigations can be reconciled, considering that SASP pro-growth effects were observed when senescent cells were cultivated in the presence of cancer cells or were injected *in vivo* and wound up at tumor stroma. In both conditions, senescent cells were primed by cancer cells that may coax senescent cells to secrete factors for their growth and survival.

In a previous finding, we demonstrated that the SASP of naïve senescent cells (not primed by cancer cells) may block the proliferation and induce senescence of an immortalized lymphoblastoid cell line.

On the other hand, preliminary incubation of senescent cells with immortalized cells impair the anti-proliferative and pro-senescence activity of the SASP. This phenomenon was associated with a significant modification of SASP composition following priming with immortalized cells. Many pro-senescent and apoptotic factors present in the SASP of naïve senescent cells were absent in the secretome of primed cells [12].

Cancer stage may also have a role on the capacity of the SASP to block tumor proliferation and promote onset of the senescent phenotype. Indeed, full senescence is associated with a permanent cell cycle arrest, which needs functional cell cycle checkpoints. Following genotoxic stress with DNA damage, cell cycle checkpoints can be activated in the G_1_ phase, S phase, or G_2_/M transition phase. The activation of these checkpoints leads to cell cycle arrest to repair DNA. If DNA is mis-repaired, cells enter apoptosis or senescence. Alternatively, cancer cells with mutated/damaged DNA may still proliferate and grow in uncontrolled ways since genes that regulated checkpoints are not active [13]. In this scenario, it is of interest to evaluate how the SASP from senescent cells can cope with immortalized cells that still have partially operative cell cycle checkpoints and metastatic cancer cells that have completely deregulated checkpoints. We decided to prove this research hypothesis by treating immortalized prostate and metastatic prostatic cancer cell lines with the SASP from naïve mesenchymal stromal cells (MSCs) to evaluate the effect on proliferation, apoptosis, and senescence. We chose MSCs since stromal cells are an integral part of the cancer microenvironment and are involved in tumor proliferation, angiogenesis, invasion, and metastasis [14].

## RESULTS

We aimed to evaluate the SASP effect on the *in vitro* biological properties of PNT2 and PC3 cell lines, which are immortalized prostate cells and metastatic prostatic cancer cells, respectively. Cells of PNT2 cell line were immortalized by infection with the SV40 virus. The large T antigen of the SV40 virus blocks RB1 and P53 proteins, thus impairing the cell cycle checkpoints [15]. PC3 cells have dozens of mutations in genes involved in cell cycle regulation [16]. After extensive culturing (30 days *in vitro*), the MSCs reached replicative senescence, and we collected the SASP from these chronic senescent cells. We induced acute senescence of MSCs by treatment with H_2_0_2_, and in this case, we collected the SASP. Then we incubated PNT2 and PC3 cells with SASPs from acute and chronic senescent cells, A-SASP and C-SASP, respectively. Cells were incubated for 48 hours with SASPs for all the experiments, but colony soft agar assay. For this latter experiment the incubation of cells with SASPs was longer (see Methods).

**Figure 1:**
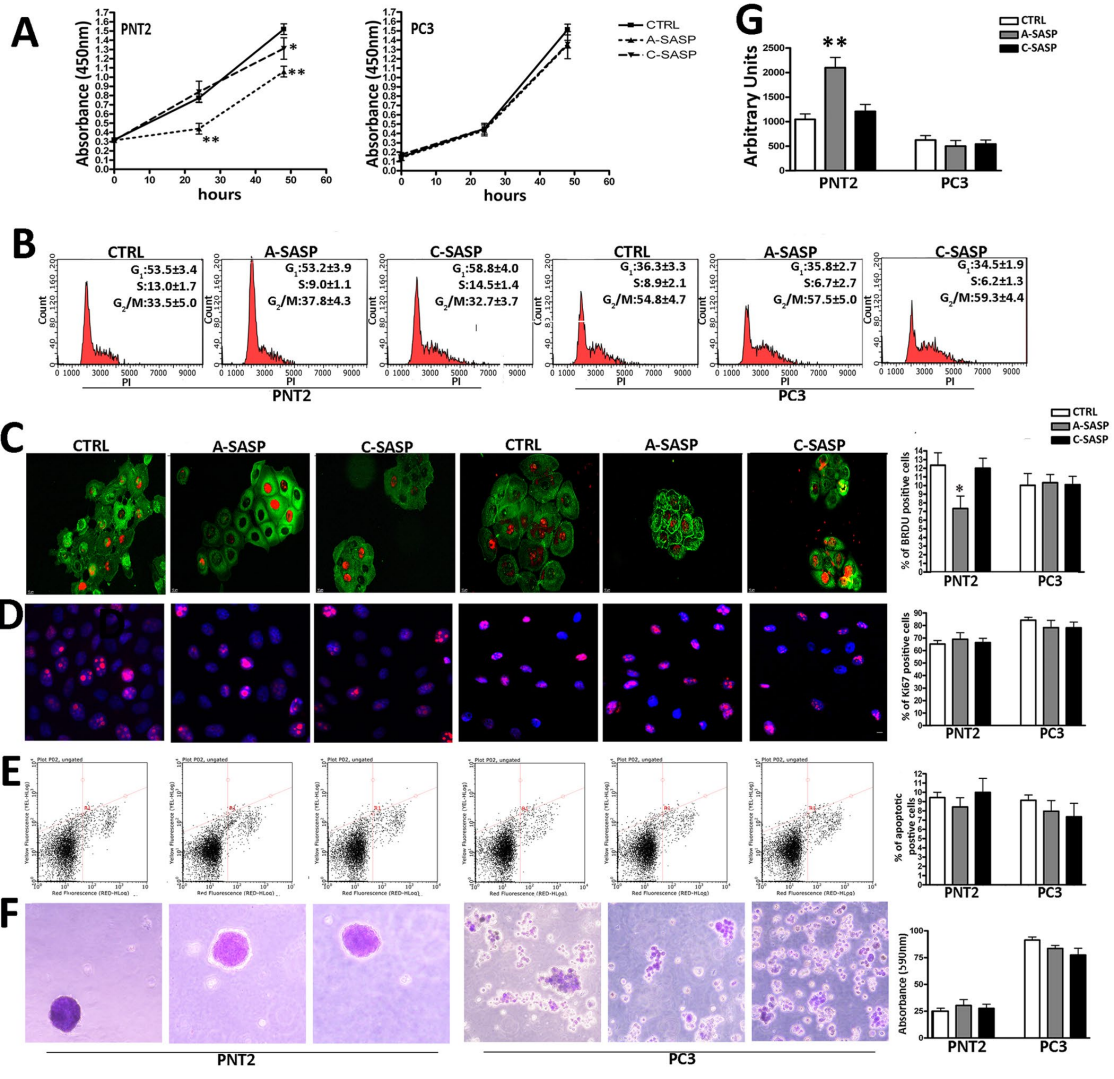
**Responsiveness of immortalized and metastatic cancer cells to A-SASP from senescent MSCs.** (**A**) PNT2 and PC3 cell proliferation was determined by Cell Counting Kit-8 (CCK-8) colorimetric assay (Dojindo, Germany). On the left, PNT2 proliferation in control medium (CTRL) and in media containing either an A-SASP or C-SASP. On the right, PC3 proliferation (data are expressed with standard deviation SD, n = 3, *p < 0.05; **p < 0.01). (**B**) Representative cell cycle FACS analysis of PNT2 and PC3 cultures treated with SASPs. (n=3 ± SD). (**C**) Representative micrographs of BrdU immunostaining (red) on PNT2 and PC3 cultures treated with SASPs. Cell cytoplasms were stained with actin (green). The graph shows the percentage of cycling (BrdU-positive) PNT2 and PC3 cells in the presence of different SASPs (n = 3 ± SD, *p < 0.05). (**D**) Representative micrographs of Ki67 immunostaining (red) on PNT2 and PC3 cultures treated with SASPs. Cell nuclei were stained with DAPI (blue). The graph shows the percentage of cycling (Ki-67-positive) PNT2 and PC3 cells in the presence of different SASPs (n = 3 ± SD). (**E**) Representative apoptosis FACS analysis. The experiments were carried out after treatment with SASPs. The assay allows the identification of early (Annexin V + and 7ADD −) and late apoptosis (Annexin V + and 7ADD +). The histogram shows the global percentage of Annexin V-positive cells. Data are expressed with standard deviation (n = 3 ± SD). (**F**) Representative micrographs of colony in suspension from PNT2 and PC3 cultures treated with SASPs. Colonies were identified by crystal violet staining. The table shows the 590 nm absorbance of crystal violet released by colonies after de-staining samples in 100% methanol (n = 3 ± SD) [26]. (**G**) MUG quantitative senescence assay in PNT2 and PC3 cultures. The graph shows mean percentage value of senescence. 4-MUG is a beta-galactosidase substrate that does not emit fluorescence until cleaved by the enzyme to generate the fluorophore 4-methylumbelliferone. In the different experimental conditions, weperformed an assay on cell lysates to monitor the fluorophore production; the results are shown in the graph and are expressed as arbitrary units (n = 3 ± SD **p< 0.01).

The proliferation assay evidenced that the A-SASP was effective in reducing proliferation in PNT2 but not in PC3 cells (Figure 1A). The C-SASP decreased PNT2 cell proliferation even though to a lesser extent if compared to the A-SASP. The C-SASP was ineffective on PC3 cell proliferation (Figure 1A). A cell cycle analysis of PNT2 cells evidenced that only the A-SASP was able to reduce the number of S-phase cells (Figure 1B). The cell cycle profile of PC3 was unaffected by both SASPs (Figure 1B). Data of cells in S phase was confirmed with BrdU immunostaning. We performed a two hours pulse of PNT2 and PC3 cells with BrdU and then determined the percentage of positive cells in the different experimental conditions. A-SASP reduced the percentage PNT2 cells in S-phase cells compared with controls (7.7% ± 0.9% vs 12% ± 1.2%) while C-SASP was ineffective (12.7% ± 1.9% vs 12% ± 1.2%) (Figure 1C). We detected 9.8% BrdU(+) PC3 cells in control cultures and this percentage was not significantly affected by treatment with SASPs (Figure 1C). The percentage of Ki67(+) cycling cells was not influenced by SASP treatments (Figure 1D). Both the A- and C-SASP did not modify the apoptosis levels in PNT2 and PC3 cells (Figure 1E). Of interest, A-SASP induced senescence in PNT2 cells while it did not affect PC3 cells (Figure 1G). Tumorigenicity of both PNT2 and PC3 cells was not modified by SASP treatment as evidenced by the soft-agar assay (Figure 1F).

The higher effectiveness of the A-SASP compared with the C-SASP in reducing proliferation and triggering senescence phenomena in prostate immortalized cells was confirmed by treating PNT2 cells with the secretome of acutely X-ray-irradiated MSCs (Supplementary Figure 1).

In some experimental models, SASPs from naïve senescent cells negatively regulated proliferation and induced senescence or apoptosis of immortalized cell lines [12]. In this study, we observed only a partial response of PNT2 cells to the SASP with minimal effect on proliferation and senescence, while PC3 cells were not affected by treatment with senescent secretomes. To better understand this phenomenon, we performed an evaluation of proteins that are the master regulators of cell cycle, senescence, and apoptosis.

**Figure 2:**
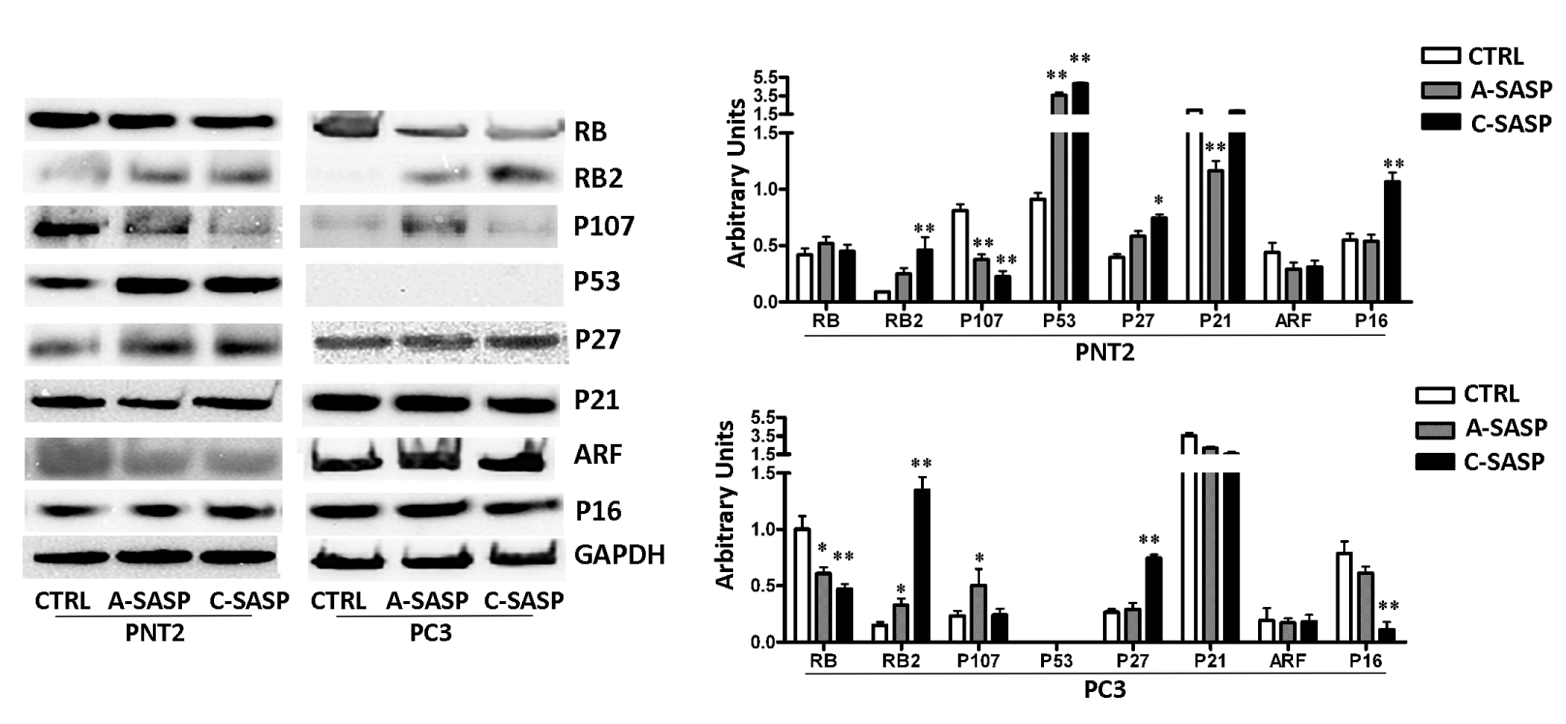
**Western blot analysis of an SASP effects the SASP on immortalized and metastatic cancer cells.** The picture shows the expression levels of proteins involved in regulation of cell cycle, senescence, and apoptosis. GAPDH was used as a loading control. The graph shows mean expression levels, n = 3 ±SD, *p < 0.05, **p < 0.01).

PNT2 cells treated with the A-SASP showed a significant increase in P53 and RB2/P130 proteins, members of the retinoblastoma (RB) gene family (Figure 2). The expression of P107, another member of the RB family, which is expressed mainly in cycling cells, decreased significantly (Figure 2). Only P27KIP1 among the analyzed cyclin-dependent kinase inhibitors (CDKIs) evidenced a significant upregulation compared to control cultures (Figure 2). Of note, incubation of PNT2 cells with the C-SASP produced higher upregulation of proteins involved in cells cycle exit, senescence, and apoptosis compared with the A-SASP (Figure 2), despite this latter SASP inducing more evident biological phenomena. In PNT2 cultures, the C-SASP induced a strong increase in P53 and RB2/P130 protein levels. This phenomenon was associated with a decrease of P107 and an upregulation of P27KIP1 and P16 (Figure 2).

Also, the treatment of PC3 with SASPs promoted changes in the expression of the proteins involved in the control of cell proliferation, cell death, and senescence. In detail, in cells treated with the A-SASP, we observed a decrease of RB1 and an augmentation of P107 and RB2/P130. These changes appear at odds since P107 is mainly expressed in cycling cells while RB2/P130 is upregulated in quiescent/senescent cells [17] (Figure 2). Similar contrasting expression profiles were observed in PC3 incubated with the C-SASP (Figure 2).

Collectively our data, showed that pre-tumorigenic cells were SASP responsive, while cancer cells were not. We then performed a preliminary analysis to evaluate if cancer cells with various grades of malignancy may show difference in SASP sensitivity. In prostate cancer the low androgen sensitivity is associated with a high malignant phenotype. PC3 cells are androgen-independent prostate cancer cell lines and hence their unresponsiveness to SASP treatment may be related to their high malignancy [18]. Indeed, also treatment of androgen sensitive LNCaP cancer cells [19] with either A-SASP or C-SASP did not produce significant changes in proliferation, apoptosis and senescence (Supplementary Figure 2).

### A-SASPs appeared more effective than C-SASPs in triggering senescence of immortalized cells

**Figure 3:**
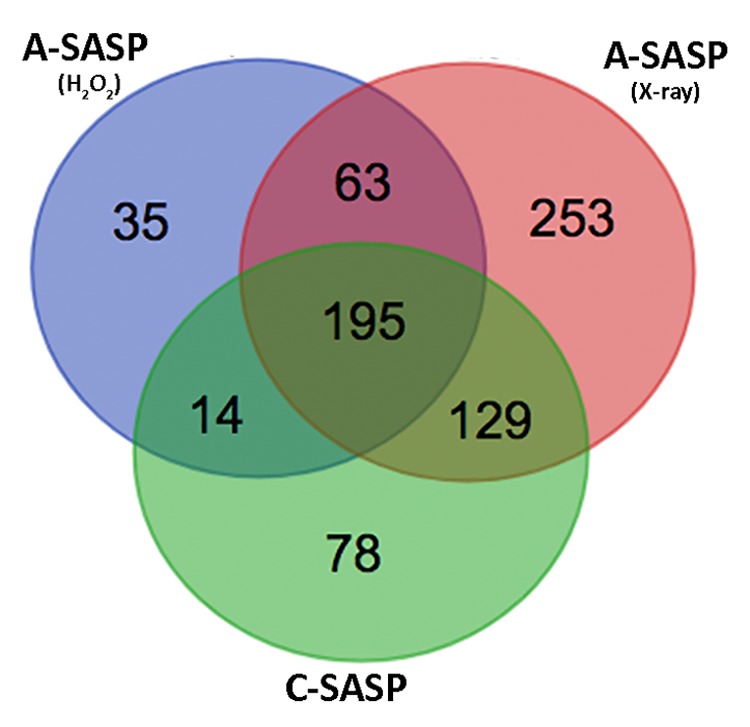
**Comparison of SASP contents.** Venn diagram showing common and specific proteins among secretomes obtained from X-ray-irradiated or H_2_0_2_-treated or senescent-replicative MSCs.

Our data evidenced that A-SASPs, coming either from cells treated with H_2_0_2_ or with low X-ray doses, induced senescence to a higher extent compared with C-SASPs. We decided to analyze more in depth the origin of the observed difference in biological performances. To this end, we evaluated publicly available data on proteome compositions of SASPs from acute and chronic senescent MSCs we published [4]. In previous research we performed LC-MS/MS proteome identification of A- and C-SASPs. We found 416 proteins in the C-SASP, 307 proteins in the SASP of H_2_0_2_-treated MSCs, and 640 proteins in the SASP of X-ray-irradiated cells. In this current study, we used a Venn diagram to identify common and specific factors among these SASPs (Figure 3). We identified 63 proteins present only in the A-SASP and not in the C-SASP (Supplementary File 1). We reasoned that these proteins may account for better effectiveness of the A-SASP compared to the C-SASP in inducing senescence of immortalized cells. We then classified these 63 proteins through gene ontology (GO) analysis by using Panther software. Panther Protein class analysis found 12 proteins belonging to the chaperonin class (Supplementary File 2). In the Panther GO Molecular Functions dataset, we identified several ontological classes, such as: Unfolded protein binding, Cadherin binding, RNA binding, and ATP binding (Supplementary File 3). In the Panther Reactome pathway dataset, we noticed several pathway-related classes, such as: Folding of actin by CCT/TriC, Metabolism, and G_2_/M transition (Supplementary File 4).

We then refined the GO analysis with DAVID software, which uses a functional annotation tool to determine relationships among similar GO annotation terms. This analysis focuses more on the biological interpretation of specific protein subsets. Functional annotation analysis gathers related GO terms into clusters that receive a group enrichment score (GES). Top-ranked GESs contain annotation members that may play a key role in the experimental conditions under analysis. The DAVID cluster with the highest enrichment score contained annotation members of chaperonin and folding protein GO (Supplementary File 4).

## DISCUSSION

For a long time, senescence has been considered an anti-cancer phenomenon since it can arrest the growth of tumor cells [20]. At odds with this statement, there are many findings showing that, in some experimental conditions, senescent cells may promote cancer growth or at least be ineffective in arresting tumor cell proliferation [12,20]. These findings are apparently conflicting if complexity of senescence is not taken in account. Senescent cells are not all equal; they have both common and unique features depending on cell type and genotoxic agents that trigger senescence [3,4]. Besides, two other aspects of senescence have to be considered. Interaction of senescent cells with immortalized/malignant cells may impair or even abrogate the anti-tumor activity of the SASP. Finally, the target cells that receive signals from senescent cells through the SASP must have functional cell cycle checkpoints in order to enter permanent arrest and senescence.

### SASPs arrested the growth of immortalized cells and induced senescence but were ineffective on cancer cells

In our previous findings, we addressed some of the issues described above. We analyzed biological features of chronic senescent cells (replicative exhaustion) and acute senescent cells, obtained by oxidative stress, doxorubicin treatment, and X-ray irradiation. The different senescent phenotypes, besides common features, showed specific changes in autophagy, proteasome activity, metabolism, and SASP composition [3,4]. In another investigation, we demonstrated that naïve senescent MSCs arrested proliferation and induced senescence in ARH77 cells, an immortalized lymphoblastoid cell line. This anti-tumor activity was impaired by preventive contact (priming) with immortalized cells [12].

In the current study, we evidenced that the SASP from acute senescent cells can block proliferation and induce senescence only in immortalized cells (PNT2) while fully cancer cells appeared insensitive to SASP antitumor activity. Incubation of PNT2 cells with the A-SASP induced a strong upregulation of several proteins involved in cell cycle exit and onset of apoptosis and/or senescence. Nevertheless, in PNT2 cells treated with the A-SASP, the percentage of cycling cells, as detected by Ki67 immunostaining, was not reduced compared with control cultures. This event suggests that cell cycle exit and ensuing senescence in PNT2 cells are unstable.

Indeed, our data indicates that cells respond to genotoxic stress trying to activate the pathways that lead to cell cycle arrest and senescence but this process was ineffective, since the two senescence-driving signaling pathways (RB and P53 paths) are impaired in PNT2 cells due to SV40 transduction. The unstable cell cycle exit hypothesis fits well with recent discoveries evidencing that Ki67 protein expression is not binary, since Ki67 is constantly degraded during G_1_ and G_0_ phases and is continuously synthesized starting from S phase until the end of mitosis. Ki67 expression is a function of cell cycle history: its level declines progressively as cells spent time in G_0_ and persists steadily in this state [21]. Our previous finding lends further credit to this hypothesis. In that studies we evidenced that A- and C-SASPs may induce permanent cell cycle exit and senescence onset in immortalized cells only if they have at least one functional, senescence-related pathway. Treatment with A- or C-SASPs of ARH77 cells, having mutated P53 and functional RB pathways, induced significant reduction of cells in the S-phase associated with a decrease of Ki-67(+) cells and a significant augmentation of senescent cells [12].

The current data and previous research are in good agreement with the theory of cancerogenesis as a multi-step process producing malignant cells, which are progressively less responsive to anti-cancer defense since they accumulate genomic mutations that completely abrogate or heavily impair cell cycle checkpoints. Indeed, malignant PC3 cells did not enter senescence or apoptosis following treatment with SASPs. This insensitivity is associated with a deregulated and mixed-up expression of proteins involved in cell cycle regulation.

The better performances of A-SASPs compared to C-SASPs are obviously related to differences in their composition. Our GO analysis of the 63 proteins present only in A-SASPs evidenced that chaperonins and factors involved in protein folding may be the most representative class of proteins that distinguishes A-SASPs from C-SASPs. Indeed, endoplasmic reticulum stress and improper protein folding are associated with senescence onset [22,23]. In this scenario, the presence in A-SASPs of factors involved in proper protein folding is counteractive to the consideration that secretome from senescent cells should promote by paracrine mechanisms the senescence of surrounding cells. Nevertheless, deregulated activity of chaperonins and other folding proteins may induce cell stress and subsequent senescence. Further investigations on the role of chaperonins in senescence are of great interest.

## MATERIALS AND METHODS

### MSC cultures

We collected bone marrow from three healthy donors who provided informed consent. Experimental protocols were approved by the Ethical Committee from Campania University “Luigi Vanvitelli. We loaded cells on a Ficoll density gradient (GE Healthcare, Italy) and then we isolated the mononuclear cell fraction. We seeded 1-2.5 × 105 cells/cm2 in α-MEM containing 10% Foetal Bovine Serum (FBS) and basic FGF. After 72 hr, the non-adherent cells were washed away and adherent cells were cultivated to confluency. Cells were then further cultivated for the experiments reported below.

### Acute and chronic senescence

For the induction of acute senescence, we used two different stressors: peroxide hydrogen and irradiation treatments. For peroxide treatment, MSCs were incubated with 300 μM H_2_O_2_ for 30 min in a complete medium. The medium was then replaced with a fresh one, and the cells were incubated for 48 hr before further analysis. For irradiation, we used exponentially growing MSCs at passage 3. These cells were irradiated with 40 mGy X-rays at room temperature. A Mevatron machine (Siemens, Italy) operating at 6 MeV was used for irradiation. After irradiation, cells were further cultivated for 48 hr before performing experiments. This time point was selected following our previous studies showing that a senescent phenotype is present 48 hr following a genotoxic stress [4,17].

Chronic senescent MSCs were obtained by extensive *in vitro* cultivation for 30 days (replicative senescence) as previously described [24].

### PNT2, PC3 and LNCaP cell cultures

PNT2 prostate immortalized epithelium cells, PC3 Caucasian prostate adenocarcinoma cells and LNCaP cancer prostatic cells were obtained from Sigma-Aldrich, Italy, and were cultivated in DMEM containing 10% FBS.

### Cell proliferation assay and cell cycle analysis

We evaluated cell proliferation with Cell Counting Kit-8 (CCK-8) colorimetric assays for the determination of cell viability in cell proliferation and cytotoxicity assays (Dojindo Molecular Technology, Japan). We seeded 5,000 cells in 96-wells and CCK-8 were added. The viability was detected by a microplate reader at 450 nm 24 hr, 48 hr, and 72 hr after the incubation.

For each cell cycle analysis, cells were harvested and fixed in 70% ethanol. Cell samples were then washed in Phosphate Buffer Saline (PBS) and finally were dissolved in a hypotonic buffer with propidium iodide. Samples were loaded on a Guava EasyCyte flow cytometer (Merck Millipore, Milano, Italy) and analyzed with a standard procedure using EasyCyte software.

### BrdU immunodetection

Cells, grown on glass coverslips, were incubated with 10 μM BrdU for 2 hr (Sigma Aldrich, MO, USA). The cells were then washed in PBS, fixed with 100% methanol at 4 °C for 10 min and then incubated with 2 N HCl for 60 min at RT. Following HCl neutralization with a 0.1 M borate buffer additional, cells were PBS washed and then incubated with an anti-BrdU mouse monoclonal antibody (clone BMC 9318, Roche, Germany) and anti-Actin rabbit polyclonal antibody (code A2066 - Sigma-Aldrich Italy). The secondary antibodies, TRITC-conjugated (A-11004) or FITC-conjugated (71-1900), were from ThermoFisher (MA, USA). Cells were observed through a fluorescence microscope (Leica Italia, Italy). The number of BrdU-positive cells was calculated by counting at least 500 cells in different microscope fields.

### Ki67 immunodetection

Ki67 was detected on cells previously fixed with 4% paraformaldehyde by mouse monoclonal anti-Ki67 antibody (sc-7846 of Santa Cruz Biotechnology, Italy) according to manufacturer’s protocols. The secondary antibody TRITC-conjugated (A-11004) was from ThermoFisher (MA, USA). Nuclear staining was performed with a DAPI mounting medium (ab104139, ABCAM, UK). Cells were observed through a fluorescence microscope (Leica Italia, Italy). The number of Ki67-positive cells was calculated by counting at least 500 cells in different microscope fields.

### Annexin V assay

We detected apoptosis by a fluorescein-conjugated Annexin V kit (Merck Millipore, Italy) on a Guava EasyCyte flow cytometer following the manufacturer’s instructions. The kit utilizes two separate dyes, Annexin V and 7AAD, to identify three cell populations: early-apoptotic cells (Annexin V+ and 7AAD-), late-apoptotic or dead cells (Annexin V+ and 7AAD+), and non-apoptotic cells (Annexin V- and 7AAD). In our experimental conditions, we grouped early- and late-apoptotic cells.

### Quantitative senescence assay

Senescence was evaluated with a quantitative senescence-associated beta-galactosidase assay. Essentially, 4-methylumbelliferyl-β-d-galactopyranoside (4-MUG) is a beta-galactosidase substrate that does not emit fluorescence until cleaved by the enzyme to generate the fluorophore 4-methylumbelliferone. As already reported, we performed an assay on cell lysates to monitor the fluorophore production at an emission/excitation wavelength of 365/460 nm [25].

### Colony soft agar assay

We used the soft Agar assay for colony formation to detect malignant transformation of cells. For this assay, we cultivated PNT2 and PC3 cells in soft agar medium for 21 days according to the published protocol [26]. Following this incubation period, formed colonies were stained with crystal violet and quantified.

### Collecting the SASP from senescent cells and treatment of PNT2 and PC3 cells

We harvested SASPs from acute and chronic senescent MSCs. In detail, 48 hr following H_2_0_2_ treatment or X-ray irradiation, MSC cultures were extensively washed with PBS and transferred to a chemically defined, serum-free culture medium for a 48-hr incubation. For the chronic SASP, after 30 days of *in vitro* cultivation, MSCs were washed with PBS and transferred to a chemically defined, serum-free culture medium for a 48-hr incubation.

Then, the SASPs were collected and used at 50% in fresh DMEM with 10% FBS for *in vitro* cultivation of PNT2 and PC3 cells. Three independent culture preparations were prepared and pooled to address biological variation.

### Western blotting

We analyzed proteins according to our previous protocol [27,28]. In brief, we lysed cells in a buffer containing 0.1% Triton for 30 min at 4 °C. Then, a 10–40 µg of each cell lysate was electrophoresed in a polyacrylamide gel and blotted onto a nitrocellulose membrane. We used the following primary antibodies: anti-RB1 (AV33212) and anti-GAPDH (G8795) from Sigma-Aldrich (MO, USA); anti-RB2/P130 (R27020) from BD Biosciences (CA, USA); anti-P27KIP1 (3686) from Cell Signaling (MA, USA); anti-P107 (sc-318), anti-P53 (sc-126), anti-P21CIP1 (sc-397), and anti-P19/ARF (sc-56334) from Santa Cruz Biotechnology (CA, USA); and anti-P16INK4A (ab54210) from ABCAM (Cambridge, UK). We detected immunoreactive signals with a horseradish peroxidase-conjugated secondary antibody (Santa Cruz Biotechnology, CA, USA) and reacted with ECL plus reagent (GE Healthcare, Italy). We conducted a semiquantitative analysis of protein levels using a gel documentation system (Bio-Rad, Milano, Italy).

### Gene Ontology (GO) analyses of proteins present in SASPs

We used a Venn diagram (http://bioinformatics.psb.ugent.be/webtools/Venn/) to combine the data of all experimental conditions in order to find the proteins present, specifically in A-SASPs and not in C-SASPs. The A-SASP-specific proteins were analyzed with Panther (http://www.pantherdb.org) and DAVID software (https://david.ncifcrf.gov).

For Panther analysis, we used the default statistics’ over-representation to compare classifications of multiple clusters of lists with a reference list to statistically identify the over- or under-representation of Panther ontologies. Significance was set to a p-value of .05.

For DAVID analysis, we used the default EASE Score Threshold (Maximum Probability). The EASE Score Threshold is a modified Fisher Exact P-Value for gene-enrichment analysis. It ranges from 0 to 1. The Fisher Exact P-Value corresponding to 0 represents perfect enrichment. As the default, we used a 0.1 EASE Score. DAVID identified clusters of GO terms by an Overall Enrichment Score that was based on the EASE Score of each term present in a given cluster. The higher the score, the more enriched.

### Statistical analysis

We evaluated statistical significance by ANOVA analysis followed by Student's t and Bonferroni's tests. We used mixed-model variance analysis for data with continuous outcomes. All data were evaluated with a GraphPad Prism version 5.01 statistical software package (GraphPad, CA, USA).

## CONCLUSIONS

Following genotoxic stress, cells may properly repair DNA and restore functionality, or they may accumulate irreversible damages that trigger either apoptosis or senescence. This latter phenomenon is considered an anti-cancer mechanism to arrest growth of cells with mutated/unrepaired DNA. Indeed, the SASP may block proliferation of cells surrounding a senescent cell. This capacity, however, can only be exerted on healthy cells or on those with minimal mutations, such as immortalized cells. Cancer cells appear mainly not responsive to the SASP. Hence, the SASP released by acute senescent cells should be considered a weapon effective against pre-tumorigenesis events rather an anti-cancer mechanism acting on malignant cells.

## Supplementary Material

Supplementary Figures

Supplementary File 1

Supplementary File 2

Supplementary File 3

Supplementary File 4
