# Supplementary material for: The senescence-associated secretory phenotype (SASP) from mesenchymal stromal cells impairs growth of immortalized prostate cells but has no effect on metastatic prostatic cancer cells
Source: Aging (Albany NY). 2019 Aug 14;11(15):5817–28. doi: 10.18632/aging.102172 (PMC6710033; doi:10.18632/aging.102172)
Supplement: Supplementary Figures [file aging-11-102172-s005.pdf]

SUPPLEMENTARY FIGURES

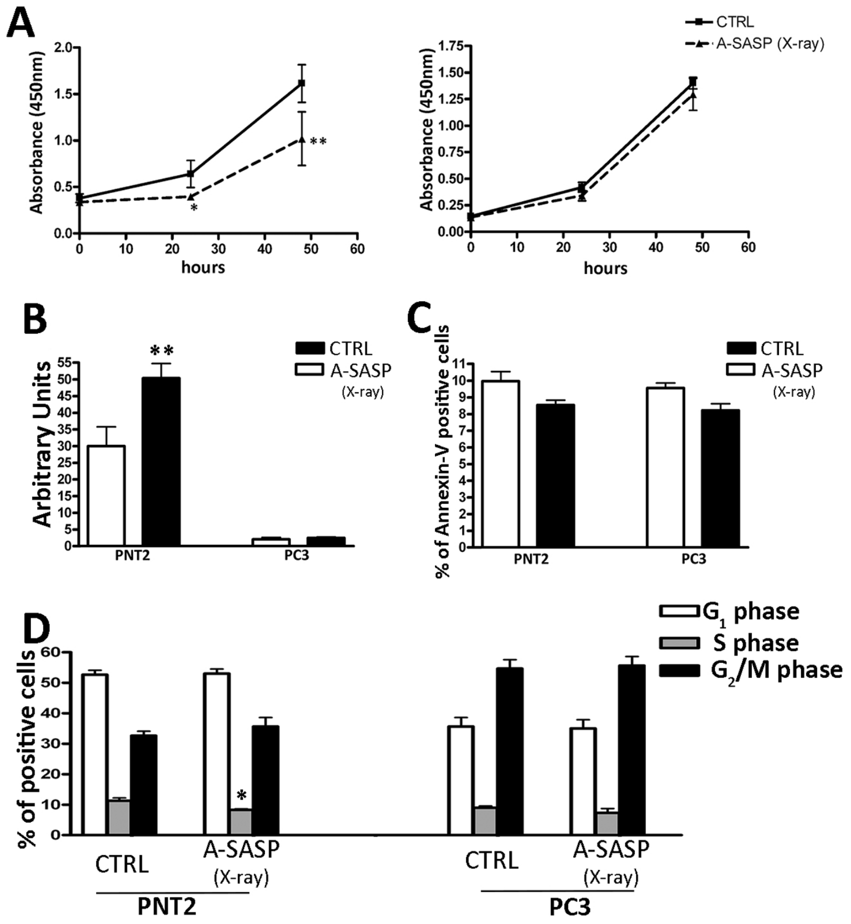

Supplementary Figure 1. Responsiveness of immortalized and metastatic cancer cells to A-SASP from irradiated MSCs. (**A**) PNT2 and PC3 cell proliferation was determined by Cell Counting Kit-8 (CCK-8) colorimetric assay (Dojindo, Germany). On the left, PNT2 proliferation in control medium (CTRL) and in media containing either an A-SASP or control medium. On the right, PC3 proliferation (n = 3 ± SD, *p < 0.05; **p < 0.01). (**B**) Senescence in PNT2 and PC3 cultures. The graph shows mean percentage value of senescent cells (n = 3 ± SD, **p< 0.01). (**C**) The histogram shows the global percentage of Annexin V-positive cells (n = 3 ± SD). (**D**) Cell cycle FACS analysis of PNT2 and PC3 cultures treated with an A-SASP (n = 3 ± SD, *p < 0.05).

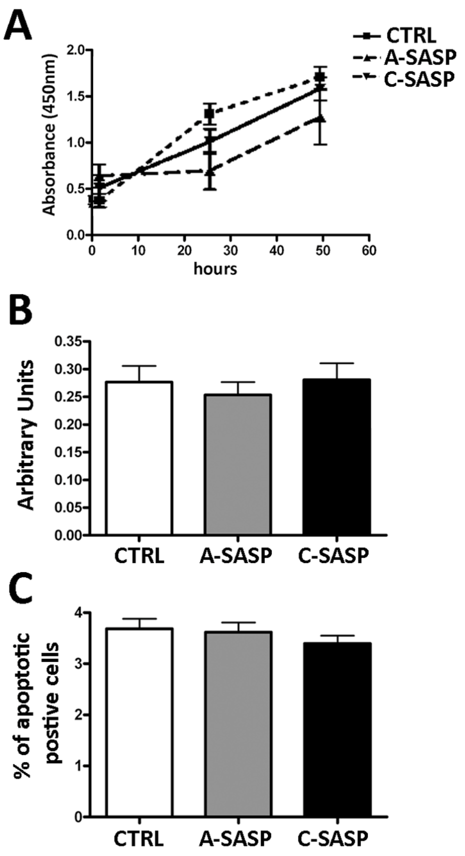

Supplementary Figure 2. Responsiveness of androgen responsive LNCaP cancer cells to SASP from senescent MSCs. (**A**) Cell proliferation was determined by Cell Counting Kit-8 (CCK-8) colorimetric assay (Dojindo, Germany). LNCaP proliferation in control medium (CTRL) and in media containing either an A-SASP or a C-SASP. (**B**) Senescence in LNCaP cultures. The graph shows mean percentage value of senescent cells (n = 3 ± SD). Data are expressed as arbitrary units. (**C**) The histogram shows the global percentage of Annexin V-positive cells (n = 3 ± SD).
