# Supplementary material for: The senescence-associated secretory phenotype (SASP) from mesenchymal stromal cells impairs growth of immortalized prostate cells but has no effect on metastatic prostatic cancer cells
Source: Aging (Albany NY). 2019 Aug 14;11(15):5817–28. doi: 10.18632/aging.102172 (PMC6710033; doi:10.18632/aging.102172)
Supplement: Supplementary File 1 [file aging-11-102172-s004.pdf]

UPL14.0 New! PANTHER14.0 is generated from the 2018_04 release of ReferenceProteome dataset

PANTHER GENE LIST

Customize Gene list

Convert List to: -Select- Send list to: -Select-

Display: 100 items per page Refine Search

Hits 1-63 of 63 [page: (1)] Number of mapped ids found 63

clr all		Gene ID	Mapped IDs	Gene Name<br>Gene Symbol<br>Ortholog	PANTHER Family/Subfamily	PANTHER Protein Class
	1.	HUMAN|HGNC=12833|UniProtKB=P13010	P13010	X-ray repair cross-complementing protein 5<br>XRCC5<br>ortholog	X-RAY REPAIR CROSS-COMPLEMENTING PROTEIN 5 (PTHR12604:SF4)	DNA helicase
	2.	HUMAN|HGNC=28833|UniProtKB=Q9NTK5	Q9NTK5	Obg-like ATPase 1<br>OLA1<br>ortholog	OBG-LIKE ATPASE 1 (PTHR23305:SF11)	G-protein
	3.	HUMAN|HGNC=9355|UniProtKB=P30044	P30044	Peroxiredoxin-5, mitochondrial<br>PRDX5<br>ortholog	PEROXIREDOXIN-5, MITOCHONDRIAL (PTHR10430:SF16)	-
	4.	HUMAN|HGNC=14678|UniProtKB=Q96CX2	Q96CX2	BTB/POZ domain-containing protein KCTD12<br>KCTD12<br>ortholog	BTB/POZ DOMAIN-CONTAINING PROTEIN KCTD12 (PTHR14499:SF29)	enzyme modulator
	5.	HUMAN|HGNC=5244|UniProtKB=P38646	P38646	Stress-70 protein, mitochondrial<br>HSPA9<br>ortholog	STRESS-70 PROTEIN, MITOCHONDRIAL (PTHR19375:SF184)	-
	6.	HUMAN|HGNC=3594|UniProtKB=P49327	P49327	Fatty acid synthase<br>FASN<br>ortholog	FATTY ACID SYNTHASE (PTHR43775:SF7)	-
	7.	HUMAN|HGNC=9530|UniProtKB=P25786	P25786	Proteasome subunit alpha type-1<br>PSMA1<br>ortholog	PROTEASOME SUBUNIT ALPHA TYPE-RELATED (PTHR11599:SF12)	protease
	8.	HUMAN|HGNC=10363|UniProtKB=P18124	P18124	60S ribosomal protein L7<br>RPL7<br>ortholog	60S RIBOSOMAL PROTEIN L7 (PTHR11524:SF12)	ribosomal protein
	9.	HUMAN|HGNC=9723|UniProtKB=P11216	P11216	Glycogen phosphorylase, brain form<br>PYGB<br>ortholog	GLYCOGEN PHOSPHORYLASE, BRAIN FORM (PTHR11468:SF3)	-
	10.	HUMAN|HGNC=9559|UniProtKB=Q13200	Q13200	26S proteasome non-ATPase regulatory subunit 2<br>PSMD2<br>ortholog	26S PROTEASOME NON-ATPASE REGULATORY SUBUNIT 2 (PTHR10943:SF15)	enzyme modulator
	11.	HUMAN|HGNC=3719|UniProtKB=Q00688	Q00688	Peptidyl-prolyl cis-trans isomerase FKBP3<br>FKBP3<br>ortholog	PEPTIDYL-PROLYL CIS-TRANS ISOMERASE FKBP3 (PTHR46493:SF1)	-
	12.	HUMAN|HGNC=10381|UniProtKB=P04843	P04843	Dolichyl-diphosphooligosaccharide--protein glycosyltransferase subunit 1<br>RPN1<br>ortholog	DOLICHYL-DIPHOSPHOOLIGOSACCHARIDE--PROTEIN GLYCOSYLTRANSFERASE SUBUNIT 1 (PTHR21049:SF0)	glycosyltransferase
	13.	HUMAN|HGNC=4162|UniProtKB=P41250	P41250	Glycine--tRNA ligase<br>GARS<br>ortholog	GLYCINE--TRNA LIGASE (PTHR10745:SF0)	aminoacyl-tRNA synthetase
	14.	HUMAN|HGNC=6715|UniProtKB=Q14767	Q14767	Latent-transforming growth factor beta-binding protein 2<br>LTBP2<br>ortholog	LATENT-TRANSFORMING GROWTH FACTOR BETA-BINDING PROTEIN 2 (PTHR24034:SF49)	annexin<br>calmodulin<br>cell adhesion molecule<br>extracellular matrix glycoprotein<br>extracellular matrix structural protein<br>signaling molecule
	15.	HUMAN|HGNC=7160|UniProtKB=P50281	P50281	Matrix metalloproteinase-14<br>MMP14<br>ortholog	MATRIX METALLOPROTEINASE-14 (PTHR10201:SF24)	metalloprotease
	16.	HUMAN|HGNC=18429|UniProtKB=Q14914	Q14914	Prostaglandin reductase 1<br>PTGR1<br>ortholog	PROSTAGLANDIN REDUCTASE 1 (PTHR43205:SF31)	dehydrogenase<br>reductase
	17.	HUMAN|HGNC=8891|UniProtKB=P52209	P52209	6-phosphogluconate dehydrogenase, decarboxylating<br>PGD<br>ortholog	6-PHOSPHOGLUCONATE DEHYDROGENASE, DECARBOXYLATING (PTHR11811:SF53)	dehydrogenase
	18.	HUMAN|HGNC=1620|UniProtKB=P40227	P40227	T-complex protein 1 subunit zeta<br>CCT6A<br>ortholog	T-COMPLEX PROTEIN 1 SUBUNIT ZETA (PTHR11353:SF54)	chaperonin
	19.	HUMAN|HGNC=1481|UniProtKB=P04632	P04632	Calpain small subunit 1<br>CAPNS1<br>ortholog	CALPAIN SMALL SUBUNIT 1 (PTHR46735:SF1)	-
	20.	HUMAN|HGNC=2898|UniProtKB=P09622	P09622	Dihydrolipoyl dehydrogenase, mitochondrial<br>DLD<br>ortholog	DIHYDROLIPOYL DEHYDROGENASE, MITOCHONDRIAL (PTHR22912:SF151)	dehydrogenase<br>oxidase<br>reductase
	21.	HUMAN|HGNC=1615|UniProtKB=P78371	P78371	T-complex protein 1 subunit beta<br>CCT2	T-COMPLEX PROTEIN 1 SUBUNIT BETA (PTHR11353:SF23)	chaperonin

			ortholog		
22.	HUMAN|HGNC=544|UniProtKB=P08133	P08133	Annexin A6<br>ANXA6<br>ortholog	ANNEXIN A6 (PTHR10502:SF177)	-
23.	HUMAN|HGNC=8766|UniProtKB=Q8WUM4	Q8WUM4	Programmed cell death 6-interacting protein<br>PDCD6IP<br>ortholog	PROGRAMMED CELL DEATH 6-INTERACTING PROTEIN (PTHR23030:SF22)	transmembrane receptor regulatory/adaptor prot
24.	HUMAN|HGNC=823|UniProtKB=P25705	P25705	ATP synthase subunit alpha, mitochondrial<br>ATP5F1A<br>ortholog	ATP SYNTHASE SUBUNIT ALPHA, MITOCHONDRIAL (PTHR43089:SF7)	ATP synthase<br>DNA binding protein<br>anion channel<br>hydrolase<br>ligand-gated ion channel
25.	HUMAN|HGNC=11845|UniProtKB=Q9Y490	Q9Y490	Talin-1<br>TLN1<br>ortholog	TALIN-1 (PTHR19981:SF7)	-
26.	HUMAN|HGNC=561|UniProtKB=O95782	O95782	AP-2 complex subunit alpha-1<br>AP2A1<br>ortholog	AP-2 COMPLEX SUBUNIT ALPHA-1 (PTHR22780:SF32)	transmembrane receptor regulatory/adaptor prot
27.	HUMAN|HGNC=9910|UniProtKB=P38159	P38159	RNA-binding motif protein, X chromosome<br>RBMX<br>ortholog	RNA-BINDING MOTIF PROTEIN, X CHROMOSOME (PTHR15241:SF68)	mRNA splicing factor
28.	HUMAN|HGNC=8905|UniProtKB=P36871	P36871	Phosphoglucomutase-1<br>PGM1<br>ortholog	PHOSPHOGLUCOMUTASE-1 (PTHR22573:SF37)	-
29.	HUMAN|HGNC=1623|UniProtKB=P50990	P50990	T-complex protein 1 subunit theta<br>CCT8<br>ortholog	T-COMPLEX PROTEIN 1 SUBUNIT THETA (PTHR11353:SF78)	chaperonin
30.	HUMAN|HGNC=16998|UniProtKB=P49755	P49755	Transmembrane emp24 domain-containing protein 10<br>TMED10<br>ortholog	TRANSMEMBRANE EMP24 DOMAIN-CONTAINING PROTEIN 10 (PTHR22811:SF147)	transfer/carrier protein<br>vesicle coat protein
31.	HUMAN|Ensembl=ENSG00000198211|UniProtKB=A0A0B4J269	A0A0B4J269	Uncharacterized protein<br>unassigned<br>ortholog	TUBULIN BETA-3 CHAIN (PTHR11588:SF43)	tubulin
32.	HUMAN|HGNC=24437|UniProtKB=Q96KP4	Q96KP4	Cytosolic non-specific dipeptidase<br>CNDP2<br>ortholog	CYTOSOLIC NON-SPECIFIC DIPEPTIDASE (PTHR43270:SF11)	deacetylase<br>metalloprotease
33.	HUMAN|HGNC=6561|UniProtKB=P09382	P09382	Galectin-1<br>LGALS1<br>ortholog	GALECTIN-1 (PTHR11346:SF97)	cell adhesion molecule<br>signaling molecule
34.	HUMAN|HGNC=6692|UniProtKB=Q07954	Q07954	Prolow-density lipoprotein receptor-related protein 1<br>LRP1<br>ortholog	PROLOW-DENSITY LIPOPROTEIN RECEPTOR-RELATED PROTEIN 1 (PTHR24270:SF23)	-
35.	HUMAN|HGNC=12469|UniProtKB=P22314	P22314	Ubiquitin-like modifier-activating enzyme 1<br>UBA1<br>ortholog	SUMO-ACTIVATING ENZYME SUBUNIT 1-RELATED (PTHR10953:SF211)	ligase<br>transfer/carrier protein
36.	HUMAN|HGNC=2254|UniProtKB=Q9ULV4	Q9ULV4	Coronin-1C<br>CORO1C<br>ortholog	CORONIN-1C (PTHR10856:SF10)	non-motor actin binding protein
37.	HUMAN|HGNC=12525|UniProtKB=O60701	O60701	UDP-glucose 6-dehydrogenase<br>UGDH<br>ortholog	UDP-GLUCOSE 6-DEHYDROGENASE (PTHR11374:SF35)	-
38.	HUMAN|HGNC=8723|UniProtKB=P05154	P05154	Plasma serine protease inhibitor<br>SERPINA5<br>ortholog	PLASMA SERINE PROTEASE INHIBITOR (PTHR11461:SF274)	serine protease inhibitor
39.	HUMAN|HGNC=4057|UniProtKB=P11413	P11413	Glucose-6-phosphate 1-dehydrogenase<br>G6PD<br>ortholog	GLUCOSE-6-PHOSPHATE 1-DEHYDROGENASE (PTHR23429:SF0)	dehydrogenase
40.	HUMAN|HGNC=8907|UniProtKB=O95394	O95394	Phosphoacetylglucosamine mutase<br>PGM3<br>ortholog	PHOSPHOACETYLGLUCOSAMINE MUTASE (PTHR45955:SF1)	-
41.	HUMAN|HGNC=10488|UniProtKB=P31949	P31949	Protein S100-A11<br>S100A11<br>ortholog	PROTEIN S100-A11 (PTHR11639:SF60)	calmodulin<br>signaling molecule
42.	HUMAN|HGNC=20771|UniProtKB=P68371	P68371	Tubulin beta-4B chain<br>TUBB4B<br>ortholog	TUBULIN BETA-4B CHAIN (PTHR11588:SF247)	tubulin
43.	HUMAN|HGNC=9583|UniProtKB=P26599	P26599	Polypyrimidine tract-binding protein 1<br>PTBP1<br>ortholog	POLYPYRIMIDINE TRACT-BINDING PROTEIN 1 (PTHR15592:SF19)	-
44.	HUMAN|HGNC=10992|UniProtKB=P12236	P12236	ADP/ATP translocase 3<br>SLC25A6<br>ortholog	ADP/ATP TRANSLOCASE 3 (PTHR45635:SF13)	-
45.	HUMAN|HGNC=5269|UniProtKB=P61604	P61604	10 kDa heat shock protein, mitochondrial<br>HSPE1<br>ortholog	10 KDA HEAT SHOCK PROTEIN, MITOCHONDRIAL (PTHR10772:SF0)	chaperonin
46.	HUMAN|HGNC=1617|UniProtKB=P50991	P50991	T-complex protein 1 subunit delta<br>CCT4<br>ortholog	T-COMPLEX PROTEIN 1 SUBUNIT DELTA (PTHR11353:SF26)	-
47.	HUMAN|HGNC=9282|UniProtKB=P62140	P62140	Serine/threonine-protein phosphatase PP1-beta catalytic subunit<br>PPP1CB<br>ortholog	SERINE/THREONINE-PROTEIN PHOSPHATASE PP1-BETA CATALYTIC SUBUNIT (PTHR11668:SF414)	calcium-binding protein<br>protein phosphatase
48.	HUMAN|HGNC=13312|UniProtKB=P78417	P78417	Glutathione S-transferase omega-1<br>GSTO1<br>ortholog	GLUTATHIONE S-TRANSFERASE OMEGA-1 (PTHR43968:SF5)	-
49.	HUMAN|HGNC=11655|UniProtKB=P17987	P17987	T-complex protein 1 subunit alpha<br>TCP1	T-COMPLEX PROTEIN 1 SUBUNIT ALPHA (PTHR11353:SF195)	chaperonin

				ortholog		
☐	50.	HUMAN|HGNC=9256|UniProtKB=P45877	P45877	Peptidyl-prolyl cis-trans isomerase C<br>PPIC<br>ortholog	PEPTIDYL-PROLYL CIS-TRANS ISOMERASE C (PTHR11071:SF11)	-
☐	51.	HUMAN|HGNC=3015|UniProtKB=Q14195	Q14195	Dihydropyrimidinase-related protein 3<br>DPYSL3<br>ortholog	DIHYDROPYRIMIDINASE-RELATED PROTEIN 3 (PTHR11647:SF57)	metalloprotease
☐	52.	HUMAN|HGNC=9846|UniProtKB=P62826	P62826	GTP-binding nuclear protein Ran<br>RAN<br>ortholog	GTP-BINDING NUCLEAR PROTEIN RAN (PTHR24071:SF22)	small GTPase
☐	53.	HUMAN|HGNC=3213|UniProtKB=P26641	P26641	Elongation factor 1-gamma<br>EEF1G<br>ortholog	ELONGATION FACTOR 1-GAMMA (PTHR43986:SF4)	-
☐	54.	HUMAN|HGNC=6904|UniProtKB=P31153	P31153	S-adenosylmethionine synthase isoform type-2<br>MAT2A<br>ortholog	S-ADENOSYLMETHIONINE SYNTHASE ISOFORM TYPE-2 (PTHR11964:SF12)	nucleotidyltransferase
☐	55.	HUMAN|HGNC=16919|UniProtKB=Q99536	Q99536	Synaptic vesicle membrane protein VAT-1 homolog<br>VAT1<br>ortholog	SYNAPTIC VESICLE MEMBRANE PROTEIN VAT-1 HOMOLOG (PTHR44054:SF1)	-
☐	56.	HUMAN|HGNC=1622|UniProtKB=Q99832	Q99832	T-complex protein 1 subunit eta<br>CCT7<br>ortholog	T-COMPLEX PROTEIN 1 SUBUNIT ETA (PTHR11353:SF22)	chaperonin
☐	57.	HUMAN|HGNC=30646|UniProtKB=Q7KZF4	Q7KZF4	Staphylococcal nuclease domain-containing protein 1<br>SND1<br>ortholog	STAPHYLOCOCCAL NUCLEASE DOMAIN-CONTAINING PROTEIN 1 (PTHR12302:SF2)	nucleic acid binding transcription cofactor
☐	58.	HUMAN|HGNC=15461|UniProtKB=P55145	P55145	Mesencephalic astrocyte-derived neurotrophic factor<br>MANF<br>ortholog	MESENCEPHALIC ASTROCYTE-DERIVED NEUROTROPHIC FACTOR (PTHR12990:SF10)	-
☐	59.	HUMAN|HGNC=8574|UniProtKB=P43034	P43034	Platelet-activating factor acetylhydrolase IB subunit alpha<br>PAFAH1B1<br>ortholog	PLATELET-ACTIVATING FACTOR ACETYLHYDROLASE IB SUBUNIT ALPHA (PTHR44129:SF6)	-
☐	60.	HUMAN|HGNC=9785|UniProtKB=P51148	P51148	Ras-related protein Rab-5C<br>RAB5C<br>ortholog	RAS-RELATED PROTEIN RAB-5C (PTHR24073:SF366)	-
☐	61.	HUMAN|HGNC=16991|UniProtKB=Q07065	Q07065	Cytoskeleton-associated protein 4<br>CKAP4<br>ortholog	CYTOSKELETON-ASSOCIATED PROTEIN 4 (PTHR45161:SF1)	-
☐	62.	HUMAN|HGNC=30688|UniProtKB=Q86VP6	Q86VP6	Cullin-associated NEDD8-dissociated protein 1<br>CAND1<br>ortholog	CULLIN-ASSOCIATED NEDD8-DISSOCIATED PROTEIN 1 (PTHR12696:SF1)	transcription factor
☐	63.	HUMAN|HGNC=16931|UniProtKB=Q9Y4L1	Q9Y4L1	Hypoxia up-regulated protein 1<br>HYOU1<br>ortholog	HYPOXIA UP-REGULATED PROTEIN 1 (PTHR45639:SF3)	-

Hits 1-63 of 63 [page: (1)]

© Copyright 2019 Paul Thomas All Rights Reserved.
