# Supplementary material for: The senescence-associated secretory phenotype (SASP) from mesenchymal stromal cells impairs growth of immortalized prostate cells but has no effect on metastatic prostatic cancer cells
Source: Aging (Albany NY). 2019 Aug 14;11(15):5817–28. doi: 10.18632/aging.102172 (PMC6710033; doi:10.18632/aging.102172)
Supplement: Supplementary File 2 [file aging-11-102172-s002.pdf]

GENEONTOLOGY Unifying Biology
PANTHER Classification System
LOGIN REGISTER CONTACT US
Home | About | PANTHER Data | PANTHER Tools | Workspace | Downloads | Help/Tutorial
UPL14.0 New! PANTHER14.0 is generated from the 2018_04 release of ReferenceProteome dataset

PANTHER GENE LIST

Customize Gene list

Convert List to: -Select- Send list to: -Select-

Display: 30 items per page Refine Search

Hits 1-12 of 12 [page: (1)] Number of mapped ids found 12

Species Filter: All

clr all		Gene ID	Mapped IDs	Gene Name Gene Symbol Ortholog	PANTHER Family/Subfamily	PANTHER Protein Class	Species
	1.	HUMAN|HGNC=11655|UniProtKB=P17987	HUMAN|HGNC=11655|UniProtKB=P17987	T-complex protein 1 subunit alpha TCP1 ortholog	T-COMPLEX PROTEIN 1 SUBUNIT ALPHA (PTHR11353:SF195)	chaperonin	Homo sapiens
	2.	HUMAN|HGNC=1615|UniProtKB=P78371	HUMAN|HGNC=1615|UniProtKB=P78371	T-complex protein 1 subunit beta CCT2 ortholog	T-COMPLEX PROTEIN 1 SUBUNIT BETA (PTHR11353:SF23)	chaperonin	Homo sapiens
	3.	HUMAN|HGNC=5269|UniProtKB=P61604	HUMAN|HGNC=5269|UniProtKB=P61604	10 kDa heat shock protein, mitochondrial HSPE1 ortholog	10 KDA HEAT SHOCK PROTEIN, MITOCHONDRIAL (PTHR10772:SF0)	chaperonin	Homo sapiens
	4.	HUMAN|HGNC=1622|UniProtKB=Q99832	HUMAN|HGNC=1622|UniProtKB=Q99832	T-complex protein 1 subunit eta CCT7 ortholog	T-COMPLEX PROTEIN 1 SUBUNIT ETA (PTHR11353:SF22)	chaperonin	Homo sapiens
	5.	HUMAN|HGNC=1621|UniProtKB=Q92526	HUMAN|HGNC=1621|UniProtKB=Q92526	T-complex protein 1 subunit zeta-2 CCT6B ortholog	T-COMPLEX PROTEIN 1 SUBUNIT ZETA-2 (PTHR11353:SF58)	chaperonin	Homo sapiens
	6.	HUMAN|HGNC=11579|UniProtKB=O75347	HUMAN|HGNC=11579|UniProtKB=O75347	Tubulin-specific chaperone A TBCA ortholog	TUBULIN-SPECIFIC CHAPERONE A (PTHR21500:SF3)	chaperonin	Homo sapiens
	7.	HUMAN|HGNC=1623|UniProtKB=P50990	HUMAN|HGNC=1623|UniProtKB=P50990	T-complex protein 1 subunit theta CCT8 ortholog	T-COMPLEX PROTEIN 1 SUBUNIT THETA (PTHR11353:SF78)	chaperonin	Homo sapiens
	8.	HUMAN|HGNC=32153|UniProtKB=A6NM43	HUMAN|HGNC=32153|UniProtKB=A6NM43	Putative T-complex protein 1 subunit theta-like 1 CCT8L1P ortholog	T-COMPLEX PROTEIN 1 SUBUNIT THETA-LIKE 1-RELATED (PTHR11353:SF100)	chaperonin	Homo sapiens
	9.	HUMAN|HGNC=1616|UniProtKB=P49368	HUMAN|HGNC=1616|UniProtKB=P49368	T-complex protein 1 subunit gamma CCT3 ortholog	T-COMPLEX PROTEIN 1 SUBUNIT GAMMA (PTHR11353:SF24)	chaperonin	Homo sapiens
	10.	HUMAN|HGNC=1620|UniProtKB=P40227	HUMAN|HGNC=1620|UniProtKB=P40227	T-complex protein 1 subunit zeta CCT6A ortholog	T-COMPLEX PROTEIN 1 SUBUNIT ZETA (PTHR11353:SF54)	chaperonin	Homo sapiens
	11.	HUMAN|HGNC=15553|UniProtKB=Q96SF2	HUMAN|HGNC=15553|UniProtKB=Q96SF2	T-complex protein 1 subunit theta-like 2 CCT8L2 ortholog	T-COMPLEX PROTEIN 1 SUBUNIT THETA-LIKE 1-RELATED (PTHR11353:SF100)	chaperonin	Homo sapiens

☐	**12.**	HUMAN|HGNC=1618|UniProtKB=P48643	HUMAN|HGNC=1618|UniProtKB=P48643	T-complex protein 1 subunit epsilon CCT5 ortholog	T-COMPLEX PROTEIN 1 SUBUNIT EPSILON (PTHR11353:SF186)	chaperonin	Homo sapiens

Hits 1-12 of 12 [page: (1)]

© Copyright 2019 Paul Thomas All Rights Reserved.
