# Supplementary material for: The senescence-associated secretory phenotype (SASP) from mesenchymal stromal cells impairs growth of immortalized prostate cells but has no effect on metastatic prostatic cancer cells
Source: Aging (Albany NY). 2019 Aug 14;11(15):5817–28. doi: 10.18632/aging.102172 (PMC6710033; doi:10.18632/aging.102172)
Supplement: Supplementary File 3 [file aging-11-102172-s003.pdf]

UPL14.0 New! **PANTHER14.0 is generated from the 2018_04 release of ReferenceProteome dataset**

Analysis Summary: Please report in publication

Analysis Type: PANTHER Overrepresentation Test (Released 20181113)

Annotation Version and Release Date: GO Ontology database Released 2019-01-01

Analyzed List: Client Text Box Input (Homo sapiens) Change

Reference List: Homo sapiens (all genes in database) Change

Annotation Data Set: GO molecular function complete

Test Type: (•) Fisher's Exact ( ) Binomial

Correction: (•) Calculate False Discovery Rate ( ) Use the Bonferroni correction for multiple testing ( ) No correction

Results

	Reference list	Client Text Box Input
Mapped IDs:	20996 out of 20996	56 out of 56
Unmapped IDs:	0	7
Multiple mapping information:	0	0

Export results

Displaying only results for FDR P < 0.05, click here to display all results

	Homo sapiens (REF)	Client Text Box Input (Hierarchy NEW!)					
GO molecular function complete	#	#	expected	Fold Enrichment	+/-	raw P value	FDR
phosphoglucomutase activity	5	2	.01	> 100	+	1.45E-04	2.82E-02
↳catalytic activity	6081	33	16.22	2.03	+	4.24E-06	1.53E-03
unfolded protein binding	131	10	.35	28.62	+	3.60E-12	1.69E-08
↳protein binding	11890	48	31.71	1.51	+	4.64E-06	1.55E-03
↳binding	15185	55	40.50	1.36	+	5.21E-07	2.44E-04
oxidoreductase activity, acting on the CH-OH group of donors, NAD or NADP as acceptor	123	5	.33	15.24	+	2.29E-05	5.96E-03
↳oxidoreductase activity, acting on CH-OH group of donors	134	5	.36	13.99	+	3.40E-05	7.97E-03
cadherin binding	315	6	.84	7.14	+	2.05E-04	3.56E-02
↳cell adhesion molecule binding	483	7	1.29	5.43	+	3.07E-04	4.79E-02
RNA binding	1659	22	4.42	4.97	+	9.33E-11	2.18E-07
↳nucleic acid binding	4123	23	11.00	2.09	+	2.62E-04	4.23E-02
↳organic cyclic compound binding	6145	35	16.39	2.14	+	3.53E-07	1.84E-04
↳heterocyclic compound binding	6058	35	16.16	2.17	+	2.97E-07	1.74E-04
ATP binding	1499	13	4.00	3.25	+	1.31E-04	2.68E-02
↳drug binding	1752	17	4.67	3.64	+	1.97E-06	8.40E-04
↳adenyl ribonucleotide binding	1556	13	4.15	3.13	+	1.90E-04	3.43E-02
↳adenyl nucleotide binding	1569	13	4.18	3.11	+	2.06E-04	3.45E-02
↳purine nucleotide binding	1928	17	5.14	3.31	+	7.15E-06	2.09E-03
↳nucleotide binding	2169	21	5.79	3.63	+	7.63E-08	5.96E-05
↳nucleoside phosphate binding	2170	21	5.79	3.63	+	7.69E-08	5.15E-05
↳small molecule binding	2553	23	6.81	3.38	+	5.16E-08	6.04E-05
↳purine ribonucleotide binding	1913	17	5.10	3.33	+	6.45E-06	2.01E-03
↳ribonucleotide binding	1928	17	5.14	3.31	+	7.15E-06	1.97E-03
↳carbohydrate derivative binding	2242	23	5.98	3.85	+	4.45E-09	6.94E-06
↳purine ribonucleoside triphosphate binding	1849	17	4.93	3.45	+	4.09E-06	1.60E-03
	2809	24	7.49	3.20	+	6.26E-08	5.87E-05

↳anion binding							
↳ion binding	6287	32	16.77	1.91	+	2.70E-05	6.65E-03
hydrolase activity	2723	18	7.26	2.48	+	1.69E-04	3.16E-02
Unclassified	3290	0	8.78	< 0.01	-	1.29E-04	2.89E-02

© Copyright 2019 Paul Thomas All Rights Reserved.

UPL14.0 New! **PANTHER14.0 is generated from the 2018_04 release of ReferenceProteome dataset**

Analysis Summary: Please report in publication

Analysis Type: PANTHER Overrepresentation Test (Released 20181113)

Annotation Version and Release Date: Reactome version 65 Released 2018-06-12

Analyzed List: Client Text Box Input (Homo sapiens) [Change]

Reference List: Homo sapiens (all genes in database) [Change]

Annotation Data Set: Reactome pathways

Test Type: (•) Fisher's Exact ( ) Binomial

Correction: (•) Calculate False Discovery Rate ( ) Use the Bonferroni correction for multiple testing ( ) No correction

Results

	Reference list	Client Text Box Input
Mapped IDs:	20996 out of 20996	56 out of 56
Unmapped IDs:	0	7
Multiple mapping information:	0	0

[Export results]

Displaying only results for FDR P < 0.05, click here to display all results

	Homo sapiens (REF)	Client Text Box Input (Hierarchy NEW!)					
Reactome pathways	#	#	expected	Fold Enrichment	+/-	raw P value	FDR
Folding of actin by CCT/TriC	10	6	.03	> 100	+	2.11E-12	2.31E-09
↳Cooperation of Prefoldin and TriC/CCT in actin and tubulin folding	32	7	.09	82.02	+	9.20E-12	5.04E-09
↳Chaperonin-mediated protein folding	94	7	.25	27.92	+	9.07E-09	1.99E-06
↳Protein folding	100	7	.27	26.24	+	1.36E-08	2.70E-06
↳Metabolism of proteins	2078	24	5.54	4.33	+	1.55E-10	6.78E-08
Formation of tubulin folding intermediates by CCT/TriC	25	7	.07	> 100	+	2.04E-12	4.47E-09
Prefoldin mediated transfer of substrate to CCT/TriC	27	7	.07	97.20	+	3.25E-12	2.37E-09
BBSome-mediated cargo-targeting to cilium	23	4	.06	65.20	+	7.53E-07	1.37E-04
↳Cargo trafficking to the periciliary membrane	51	4	.14	29.41	+	1.38E-05	1.90E-03
↳Cilium Assembly	200	6	.53	11.25	+	1.76E-05	2.15E-03
↳Organelle biogenesis and maintenance	292	8	.78	10.27	+	1.22E-06	2.05E-04
Association of TriC/CCT with target proteins during biosynthesis	38	6	.10	59.20	+	1.76E-09	5.50E-07
Cooperation of PDCL (PhLP1) and TRiC/CCT in G-protein beta folding	42	6	.11	53.56	+	3.03E-09	8.29E-07
Gene and protein expression by JAK-STAT signaling after Interleukin-12 stimulation	38	3	.10	29.60	+	1.77E-04	1.76E-02
↳Interleukin-12 signaling	47	3	.13	23.93	+	3.20E-04	2.69E-02
↳Interleukin-12 family signaling	57	3	.15	19.73	+	5.48E-04	4.29E-02
↳Immune System	2035	18	5.43	3.32	+	3.34E-06	5.22E-04
Phase II - Conjugation of compounds	108	4	.29	13.89	+	2.25E-04	1.97E-02
↳Metabolism	2061	17	5.50	3.09	+	1.72E-05	2.21E-03
Host Interactions of HIV factors	125	4	.33	12.00	+	3.86E-04	3.13E-02
↳HIV Infection	228	6	.61	9.87	+	3.61E-05	4.16E-03
↳Infectious disease	370	7	.99	7.09	+	6.09E-05	6.67E-03
↳Disease	1023	11	2.73	4.03	+	7.41E-05	7.73E-03
G2/M Transition	195	5	.52	9.61	+	1.90E-04	1.81E-02

↳Mitotic G2-G2/M phases	197	5	.53	9.52	+	1.99E-04	1.81E-02
Neutrophil degranulation	479	12	1.28	9.39	+	4.99E-09	1.21E-06
↳Innate Immune System	1102	13	2.94	4.42	+	5.41E-06	7.90E-04
Axon guidance	550	7	1.47	4.77	+	6.60E-04	4.98E-02
Unclassified	10452	6	27.88	.22	-	1.01E-09	3.70E-07

© Copyright 2019 Paul Thomas All Rights Reserved.
