# Supplementary material for: The senescence-associated secretory phenotype (SASP) from mesenchymal stromal cells impairs growth of immortalized prostate cells but has no effect on metastatic prostatic cancer cells
Source: Aging (Albany NY). 2019 Aug 14;11(15):5817–28. doi: 10.18632/aging.102172 (PMC6710033; doi:10.18632/aging.102172)
Supplement: Supplementary File 4 [file aging-11-102172-s001.pdf]

*** Welcome to DAVID 6.8 ***
*** If you are looking for DAVID 6.7, please visit our development site. ***

Functional Annotation Clustering

Help and Manual

Current Gene List: SASP protein content
Current Background: Homo sapiens
62 DAVID IDs

Options   Classification Stringency Medium

Rerun using options   Create Sublist

9 Cluster(s)   Download File

Annotation Cluster 1	Enrichment Score: 6.94			Count	P_Value	Benjamini
GOTERM_BP_DIRECT	positive regulation of protein localization to Cajal body	RT		6	3.0E-11	1.4E-8
INTERPRO	Chaperonin TCP-1, conserved site	RT		6	3.7E-11	9.2E-9
GOTERM_CC_DIRECT	chaperonin-containing T-complex	RT		6	4.4E-11	3.9E-9
GOTERM_BP_DIRECT	positive regulation of establishment of protein localization to telomere	RT		6	6.7E-11	1.6E-8
INTERPRO	Chaperone tailless complex polypeptide 1 (TCP-1)	RT		6	2.3E-10	2.9E-8
INTERPRO	TCP-1-like chaperonin intermediate domain	RT		6	2.3E-10	2.9E-8
INTERPRO	GroEL-like equatorial domain	RT		6	8.7E-10	7.2E-8
INTERPRO	Chaperonin Cpn60/TCP-1	RT		6	1.3E-9	7.8E-8
INTERPRO	GroEL-like apical domain	RT		6	1.3E-9	7.8E-8
GOTERM_BP_DIRECT	positive regulation of telomerase RNA localization to Cajal body	RT		6	1.6E-9	2.5E-7
GOTERM_MF_DIRECT	unfolded protein binding	RT		9	5.6E-9	5.2E-7
GOTERM_CC_DIRECT	zona pellucida receptor complex	RT		5	1.4E-8	4.2E-7
GOTERM_BP_DIRECT	positive regulation of telomere maintenance via telomerase	RT		6	1.0E-7	1.2E-5
UP_KEYWORDS	Chaperone	RT		9	1.4E-7	5.5E-6
GOTERM_BP_DIRECT	protein folding	RT		9	2.7E-7	2.6E-5
GOTERM_CC_DIRECT	cell body	RT		6	2.2E-6	4.8E-5
GOTERM_BP_DIRECT	binding of sperm to zona pellucida	RT		5	7.6E-6	6.0E-4
GOTERM_BP_DIRECT	toxin transport	RT		5	8.5E-6	5.7E-4
UP_KEYWORDS	Nucleotide-binding	RT		17	4.6E-5	1.5E-3
GOTERM_BP_DIRECT	scaRNA localization to Cajal body	RT		3	7.8E-5	4.6E-3
GOTERM_BP_DIRECT	protein stabilization	RT		6	1.3E-4	7.0E-3
UP_KEYWORDS	ATP-binding	RT		14	1.8E-4	4.2E-3
GOTERM_CC_DIRECT	microtubule	RT		7	5.9E-4	8.1E-3
GOTERM_MF_DIRECT	ATP binding	RT		15	7.3E-4	2.7E-2
GOTERM_BP_DIRECT	positive regulation of telomerase activity	RT		3	4.9E-3	1.8E-1
**Annotation Cluster 2**	**Enrichment Score: 2.99**			**Count**	**P_Value**	**Benjamini**
GOTERM_MF_DIRECT	cadherin binding involved in cell-cell adhesion	RT		8	8.1E-5	3.8E-3
GOTERM_CC_DIRECT	cell-cell adherens junction	RT		8	9.9E-5	1.5E-3
GOTERM_BP_DIRECT	cell-cell adhesion	RT		7	4.4E-4	2.1E-2
GOTERM_BP_DIRECT	viral process	RT		3	3.0E-1	1.0E0
**Annotation Cluster 3**	**Enrichment Score: 2.98**			**Count**	**P_Value**	**Benjamini**
INTERPRO	NAD(P)-binding domain	RT		7	2.4E-5	1.2E-3
INTERPRO	GroES-like	RT		4	4.1E-5	1.7E-3
UP_KEYWORDS	Oxidoreductase	RT		9	3.1E-4	6.2E-3
SMART	SM00829	RT		3	9.4E-4	3.9E-2
INTERPRO	Polyketide synthase, enoylreductase	RT		3	1.2E-3	4.1E-2
INTERPRO	Alcohol dehydrogenase, C-terminal	RT		3	1.2E-3	4.1E-2
GOTERM_BP_DIRECT	oxidation-reduction process	RT		8	5.5E-3	1.8E-1
UP_KEYWORDS	NADP	RT		4	1.8E-2	2.2E-1
GOTERM_MF_DIRECT	oxidoreductase activity	RT		4	3.6E-2	5.7E-1

Annotation Cluster	Category / Term		RT	Count	P_Value	Benjamini
**Annotation Cluster 4**	**Enrichment Score: 1.95**		G	**Count**	**P_Value**	**Benjamini**
	GOTERM_CC_DIRECT	myelin sheath	RT	7	1.2E-5	2.1E-4
	UP_KEYWORDS	Mitochondrion	RT	9	1.7E-2	2.2E-1
	GOTERM_CC_DIRECT	mitochondrial matrix	RT	5	2.4E-2	2.2E-1
	UP_SEQ_FEATURE	transit peptide:Mitochondrion	RT	4	1.8E-1	1.0E0
	UP_KEYWORDS	Transit peptide	RT	4	2.1E-1	8.0E-1
**Annotation Cluster 5**	**Enrichment Score: 0.79**		G	**Count**	**P_Value**	**Benjamini**
	GOTERM_MF_DIRECT	GTP binding	RT	5	5.0E-2	6.2E-1
	GOTERM_MF_DIRECT	GTPase activity	RT	3	2.1E-1	9.6E-1
	UP_SEQ_FEATURE	nucleotide phosphate-binding region:GTP	RT	3	2.4E-1	1.0E0
	UP_KEYWORDS	GTP-binding	RT	3	2.7E-1	8.4E-1
**Annotation Cluster 6**	**Enrichment Score: 0.33**		G	**Count**	**P_Value**	**Benjamini**
	GOTERM_MF_DIRECT	calcium ion binding	RT	6	1.2E-1	8.3E-1
	UP_SEQ_FEATURE	disulfide bond	RT	7	9.0E-1	1.0E0
	UP_KEYWORDS	Disulfide bond	RT	7	9.5E-1	1.0E0
**Annotation Cluster 7**	**Enrichment Score: 0.09**		G	**Count**	**P_Value**	**Benjamini**
	UP_KEYWORDS	Cell membrane	RT	10	6.1E-1	9.9E-1
	GOTERM_CC_DIRECT	plasma membrane	RT	11	9.1E-1	1.0E0
	UP_KEYWORDS	Membrane	RT	16	9.8E-1	1.0E0
**Annotation Cluster 8**	**Enrichment Score: 0.06**		G	**Count**	**P_Value**	**Benjamini**
	GOTERM_CC_DIRECT	endoplasmic reticulum membrane	RT	4	5.6E-1	1.0E0
	UP_KEYWORDS	Endoplasmic reticulum	RT	4	6.2E-1	9.9E-1
	UP_KEYWORDS	Membrane	RT	16	9.8E-1	1.0E0
	UP_SEQ_FEATURE	topological domain:Cytoplasmic	RT	4	1.0E0	1.0E0
	GOTERM_CC_DIRECT	integral component of membrane	RT	8	1.0E0	1.0E0
	UP_KEYWORDS	Transmembrane helix	RT	7	1.0E0	1.0E0
	UP_KEYWORDS	Transmembrane	RT	7	1.0E0	1.0E0
	UP_SEQ_FEATURE	transmembrane region	RT	6	1.0E0	1.0E0
**Annotation Cluster 9**	**Enrichment Score: 0.03**		G	**Count**	**P_Value**	**Benjamini**
	UP_KEYWORDS	Signal	RT	11	8.2E-1	1.0E0
	UP_SEQ_FEATURE	signal peptide	RT	8	9.0E-1	1.0E0
	UP_KEYWORDS	Glycoprotein	RT	10	9.4E-1	1.0E0
	UP_SEQ_FEATURE	glycosylation site:N-linked (GlcNAc...)	RT	6	1.0E0	1.0E0
	UP_SEQ_FEATURE	topological domain:Cytoplasmic	RT	4	1.0E0	1.0E0

105 terms were not clustered.
